# A cost–benefit algorithm for rapid diagnosis of tuberculosis and rifampicin resistance detection during mass screening campaigns

**DOI:** 10.1186/s12879-022-07157-0

**Published:** 2022-03-04

**Authors:** Valerie Flore Donkeng-Donfack, Jules Brice Tchatchueng-Mbougua, Ngu Njei Abanda, Suzanne Magloire Ongboulal, Yvonne Josiane Djieugoue, Yannick Kamdem Simo, Micheline Mekemnang Tchoupa, Frédéric Bekang Angui, Albert Kuate Kuate, Vincent Mbassa, Edwige Mvondo Abeng Belinga, Sara Eyangoh

**Affiliations:** 1 Mycobacteriology Unit, National Tuberculosis Reference Laboratory, Centre Pasteur du Cameroun, P.O. 1274, Yaoundé, Cameroon; 2 Epidemiology and Public Health Unit, Centre Pasteur du Cameroun, Yaoundé, Cameroon; 3 National Tuberculosis Control Program, Yaoundé, Cameroon

**Keywords:** TB-LAMP, Xpert MTB/RIF, Algorithm, Screening, Mass campaigns, Prisons, Refugee camps, Cost-effectiveness

## Abstract

**Background:**

Active tuberculosis (TB) case finding is important as it helps detect pulmonary TB cases missed by the other active screening methods. It requires periodic mass screening in risk population groups such as prisoners and refugees. Unfortunately, in these risk population groups periodic mass screening can be challenging due to lengthy turnaround time (TAT), cost and implementation constraints. The aim of this study was to evaluate a diagnostic algorithm that can reduce the TAT and cost for TB and Rifampicin resistance **(**RR) detection. The algorithm involves testing with TB-LAMP followed by Xpert MTB/RIF for positive TB-LAMP cases to diagnose TB during mass campaigns in prisons and refugee camps.

**Methods:**

The National Tuberculosis Control Program (NTCP) organized routine TB mass-screening campaigns in 34 prisons and 3 villages with refugees camps in Cameroon in 2019. TB LAMP was used for initial TB diagnosis and all TB-LAMP positive cases tested with the Xpert MTB/RIF assay to determine RR. TAT and cost benefits analysis of the combined use of TB-LAMP and Xpert MTB/RIF assays was determined and compared to the Xpert MTB/RIF assay when used only.

**Results:**

A total of 4075 sputum samples were collected from TB presumptive, 3672 cases in 34 prisons and 403 samples in 3 villages. Of the 4,075 samples screened with TB-LAMP, 135 were TB positive (3.31%) and run on the Xpert MTB/RIF. Of the 135 positives cases, Xpert MTB/RIF revealed 3 were RR (2.22%). The use of TB-LAMP followed by testing with Xpert MTB/RIF for TB and RR detection reduced the TAT by 73.23% in prisons and 74.92% in villages. In addition to a reduced TAT, the two molecular tests used in synergy is cost benefit from year 2 onwards.

**Conclusion:**

This study demonstrates the advantages of a diagnostic algorithm based on an initial testing with TB-LAMP followed by testing with Xpert MTB/RIF for TB diagnosis. This approach improved early and rapid TB detection with an added advantage of providing RR status. The proposed algorithm is effective and less costly from the second year of implementation and should be used by TB control programs.

**Supplementary Information:**

The online version contains supplementary material available at 10.1186/s12879-022-07157-0.

## Background

Tuberculosis (TB) is a leading cause of morbidity and mortality worldwide. Each year, an estimated 10 million people develop TB, and another 1.5 million die from TB [1]. TB unequally affects certain population groups such as prisoners and refugees that have limited access to health care [2–4]. The crowded living conditions of most prisons and refugee villages facilitate TB transmission [5–8]. In Addition, prisons also serve as an optimal environment for the transmission of TB to the general population through prison staff, visitors and released inmates [9]. In Cameroon, the prevalence of TB is largely unknown but estimated to be about 3500 per 100,000 prisoners [10]. Conversely, the annual TB incidence in prisons is 1700 per 100,000 prisoners and it is about 10 times higher than those of the general population (186 per 100,000 population) [11]. The burden of TB within refugee camps is rarely reported or unknown. However, according to a 2020 National TB survey, the incidence of TB in villages in close proximity to refugee camps in the East region of Cameroon was estimated at 25 per 100,000 population [10].

In response to the burden of TB in prisons and refugee camps, The National Tuberculosis Control Program of (NTCP) of Cameroon, introduced several strategies to reduce transmission. Among which, the active screening of prisoners and refugees with presumptive pulmonary TB (PTB). Active screening entails systematic screening for TB signs and symptoms at point of entry, contact tracing of exposed cases and periodic mass screenings [13, 14]. Periodic mass screening in prisons is extremely important as it helps to detect PTB cases missed by the other active screening methods. Unfortunately, periodic mass screening can be challenging and difficult to sustain due to high cost and implementation constraints [14, 15] such as overcrowded facilities, high number of sputum samples and limited laboratory capacity to process high number of samples. These implementation challenges could be mitigated by the use of highly sensitive, rapid, and cheap diagnostic kits. Molecular-based TB diagnostic kits such as Xpert^®^ MTB/RIF (Cepheid, Sunnyvale USA) and TB-LAMP (Eiken Chemical, Tokyo Japan) provides such convenience. However, there is very little evidence to guide policymakers on the relative cost-effectiveness and benefits of using these tests for mass screening across prison facilities. The Xpert MTB/RIF assay uses real-time polymerase chain reaction technology to detect MTB and rifampicin resistance (RR). The Xpert MTB/RIF assay, requires minimal expertise, and has a short turnaround time (TAT) of two hours to confirm MTB and detect rifampicin resistance [16]. The Xpert MTB/RIF assay is recommended by WHO since 2010 [17] and has been in used in Cameroon since 2012 with excellent performance. WHO has endorsed TB-LAMP in 2016 [18] and it has been implemented in Cameroon in 2017. TB-LAMP has a TAT of one hour, is easy to use, results are easy to interpret but it does not detect rifampicin resistance [18, 19]. According to several studies, including one conducted in Cameroon, TB-LAMP and Xpert MTB/RIF displayed similar sensitivity and specificity [18–21]. In this study, we sought to evaluate if a diagnostic algorithm based on an initial testing with TB-LAMP followed by testing with Xpert MTB/RIF to diagnose TB could reduce the TAT and be cost-beneficial during mass campaigns in prisons and in refugee camps compared to Xpert MTB/RIF when used alone.

## Methods

### Study setting and population

Cameroon is a central African country with a prison population of about 30,701 inmates. There are about 79 functional prisons spread-out across the country [22]. There are also several refugees’ camps in the Eastern and Northern regions of Cameroon where displaced people from neighboring countries are settled. Most of these camps are within local villages.

In 2016 Cameroon’s NTCP launched its first TB mass screening campaign targeting just one prison. By 2018, the NTCP was able to do mass screening for TB in all prisons. During the 2018 mass screening, 2428 samples were collected, and Xpert MTB/RIF assay was used for bacteriological analysis. The four-module Xpert MTB/RIF instrument was used with an testing capacity of 12–16 samples/day [23]. Using this Xpert MTB/RIF equipment, results were not timely and patient care was extensively delayed. In addition, samples were of poor quality as adequate storage conditions were non-existent. To speed up the diagnostic turnaround time, for the 2019 mass screening campaign, the NTCP recommended the use of TB-LAMP for initial testing of samples and then Xpert MTB/RIF to test the presence of resistance in TB-LAMP positive cases. With TB-LAMP, 14 specimens are processed in one run and about 70 or more samples can be processed per day [24]. As such, we sought to determine the TAT and cost of using this new diagnostic algorithm of TB-LAMP and Xpert MTB/RIF for the mass screening of TB at prisons and refugee camps.

### Data collection

In this study, we used TB mass screening data from 34 prisons and 3 villages with refugee camp in the eastern region of Cameroon that is Tongo-Gandima, Quamy and Ndokayo. During mass screening for TB, all inmates or refugees were screened for symptoms of TB such as persistent cough, weight loss, night sweats and high temperature. For each presumptive case, a questionnaire was used to record demographic information and symptoms. After this, a sputum sample was collected for bacteriological analysis. All sputum samples were transported to the closest designated TB diagnostic laboratory. Nine selected TB laboratories were involved, amongst which, 06 were equipped with TB-LAMP and Xpert MTB/RIF instruments (Diagnosis and Treatment Centers (DTCs). These were the DTCs of Ngaoundere and Maroua, the Regional TB laboratory of Bertoua, the Regional Reference Laboratory of Garoua, the Regional Reference Laboratory of Douala and the National Reference Laboratory (NRL-TB) hosted by Centre Pasteur du Cameroun.). Two DTCs were equipped with the TB-LAMP alone (DTCs of Ebolowa and Mbalmayo), and one was equipped with Xpert MTB/RIF alone (DTC of Ambam). The 02 laboratories equipped with only TB-LAMP (Ebolowa and Mbalmayo) were responsible for sending positives samples to Ambam and Centre Pasteur du Cameroun, respectively.

### Sample collection and transportation

A sputum sample of about 3 ml was collected from each presumptive TB case. In larger prison facilities, sample collection could occur over several days with about 150 samples collected per day. Collected samples were placed in a cold box and transported on the same day to the designated laboratory. Once at the laboratory, samples were stored at + 4 °C until the next day for testing.

### Laboratory tests

At the laboratory, all samples were initially tested with TB-LAMP and only TB-LAMP positive samples were subsequently tested with Xpert MTB/RIF assay to detect rifampicin resistance. Both tests were performed according to the manufacturer’s (Eiken and Cepheid, respectively) instructions [17, 18]. Briefly, for the TB-LAMP assay, 60 μL of sputum sample was pipetted into a heating tube and incubated at 90 °C for 5 min. The purified DNA was eluted from the absorbent tube and transferred into the injection cap. After mixing with lyophilized reagents, the amplification mixture was incubated at 67 °C for 40 min. The final results were interpreted using ultraviolet fluorescence detection. The TAT of TB-LAMP was 60 min, and no more than 14 specimens were processed per batch [19]. For the Xpert MTB/RIF assay, sample reagent was added in a 1:2 ratio to 2 ml of sample. The closed tube was manually agitated twice followed by a 15-min incubation period at room temperature prior to transferring 2 ml of the inactivated sample to the Xpert test cartridge. Cartridges were inserted into the four-module GeneXpert MTB/RIF instrument and the automatically generated results were read after 90 min [17, 23].

### Assessment of costs

We used the ingredient cost method as described by Levin and McEwan (2001) [24] to estimate the costing of each testing algorithms. Briefly and as shown in Additional file 1: Tables S6–S9, We itemized the resources for each algorithm and obtained their respective market prices obtained from manufacturers and available in published literature.

The remunerations of key personnel directly involved in the mass screening were obtained from the NTP. This assessment include maintenance cost through warranty extension for TB-LAMP and Xpert MTB/RIF (4-Module System). The training cost take in account for TB-LAMP and Xpert MTB/RIF was not included. Such cost is usually covered by the National Tuberculosis Program through various bilateral agreements of which the cost is rarely disclosed or when disclosed vary extensively.

The cost benefit of TB diagnosis during mass screening campaign was estimated for 03 consecutive years and calculated by the difference between the cost using only Xpert MTB/RIF assays as diagnosis tool and the cost using TB-LAMP and Xpert MTB/RIF assays in combination (Table 1). The items including cost of; equipment, shipment, UPS, printer, air conditioners, consumables (cost per cartridges, losses due to error and incorrect use), maintenance and Human resources (laboratories technicians, nurses, medical doctors, Prison staff, TB Regional coordinators, driver) were considered for cost analysis.

**Table 1:** Cost estimate (USD) per algorithm and per population for 3 years

	Cost estimate (USD) per algorithm and per population					
	Prisons			Villages		
	Year 1	Year 2	Year 3	Year 1	Year 2	Year 3
Algorithm 1: Testing with Gene Xpert MTB/RIF	87591.21	74237.89	84089.58	26284.13	7547.75	11208.91
Algorithm 2: Initial testing with TB-LAMP followed by testing with Xpert MTB/RIF	87507.41	57287.24	64596.33	29944.11	5670.38	9049.94
Difference Cost between algorithm 2 and 1	83.80	16934.43	19474.59	− 3659.98	1877.36	2158.97

### Statistical analysis

Participants will be described with respect to sociodemographic, clinical and biomedical characteristics. The description will be in terms of median and interquartile range for continuous variables, and using count and percentages for categorical data. The TAT of combined TB-LAMP and Xpert MTB/RIF assay was compared to Xpert MTB/RIF assay alone. To perform this comparison, we calculated the time used to perform TB-LAMP and Xpert MTB/RIF consecutively for initial diagnosis of TB and Rifampicin resistance. We then compared this time to the requested time if Xpert MTB/RIF would have been used only. For the analysis we considered the TAT for each test as the total time required for sample preparation and amplification. We assumed that 96 min was used for TB-LAMP test to perform a batch of 14 samples and 135 min for Xpert MTB/RIF test to perform a batch of 04 samples.

## Results

### Study population

**Fig. 1:**
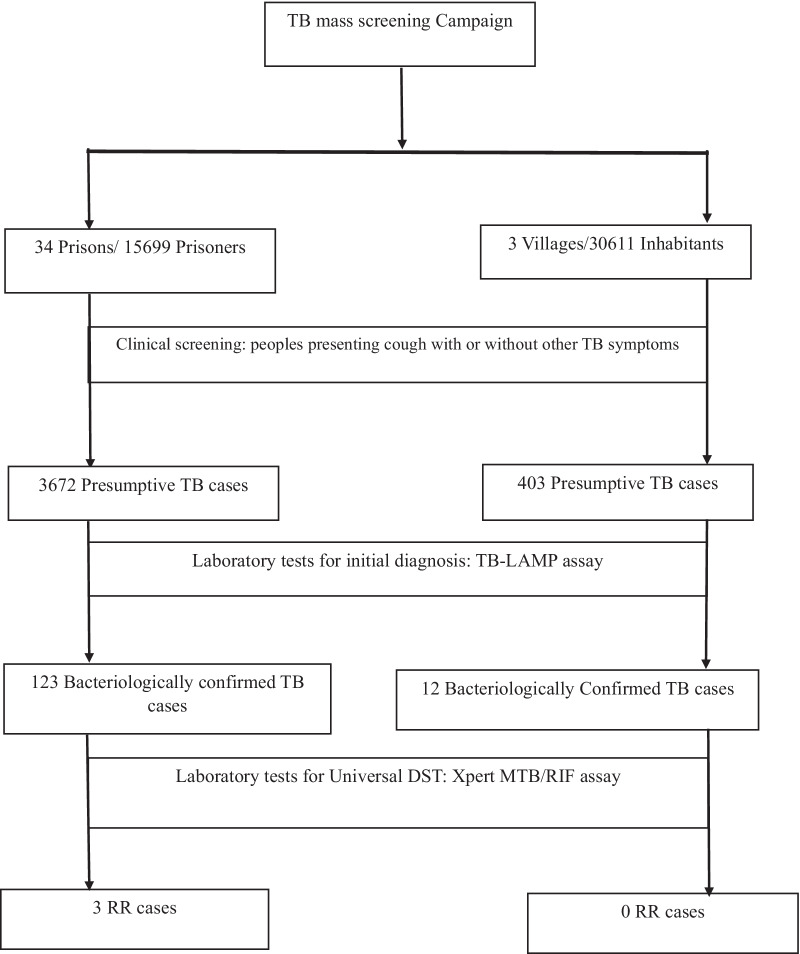
Flow chart of study design

A total of 15,699 prisoners and 30,611 villagers were screened. As shown in Fig. 1, there were 3672 presumptive TB cases among prisoners and 403 among villagers. For every presumptive TB case, about 3 ml of sputum samples were collected. The number of samples collected ranged from 27 to 483 depending on the number of prisoners at the prison facility (Table 2).

**Table 2:** Turnaround time of combined TB-LAMP and Xpert MTB/RIF test compared to Xpert MTB/RIF test used alone

Regions	Prisons	Sputum samples	TB-LAMP		Xpert MTB/RIF			Total time required for bacteriological analysis using integrated TB- LAMP and Xpert (h)	Time required for Xpert MTB/RIF analysis used alone (h)	Saved time (h)	Percentage (%)
			Time used for analysis (h)	Positive cases	Number of sputum samples	RR-TB positive cases	Time used for analysis (h)				
Adamaoua	Ngaoundéré	186	22.40	15	15	0	9	31.40	105.75	74.35	70.31
	Meigangan	70	8	4	4	0	2.25	10.25	40.50	30.25	74.69
Total	2	256	30	19	19	0	11.25	41.65	146.25	104.60	71.52
Center	Nkodengui	483	56	30	30	1	18	74.00	272.25	198.25	72.82
	Mbalmayo	139	16	5	5	0	4.5	20.50	78.75	58.25	73.97
	Bafia	45	6.40	1	1	0	2.25	8.65	27.00	18.35	67.96
	Monatele	82	9.60	6	6	0	4.5	14.10	47.25	33.15	70.16
	Eseka	27	3.20	0	0	0	0	3.20	15.75	12.55	79.68
	Sa'a	150	17.60	2	2	0	2.25	19.85	85.50	65.65	76.78
	Ntui	79	9.60	4	4	0	2.25	11.85	45.00	33.15	73.67
	Akonolinga	57	8	1	1	0	2.25	10.25	33.75	23.50	69.63
	Mfou	121	14.40	2	2	0	2.25	16.65	69.75	53.10	76.13
	Ngoumou	127	16	1	1	0	2.25	18.25	72.00	53.75	74.65
	Nanga Eboko	192	22.40	5	5	2	4.5	26.90	108.00	81.10	75.09
Total	12	1502	179.20	57	57	3	45	224.20	855.00	630.80	73.78
East	Bertoua	178	20.80	5	5	0	4.5	25.30	101.25	75.95	75.01
	Yokadouma	40	4.80	0	0	0	0	4.80	22.50	17.70	78.67
	Messamena	48	6.40	0	0	0	0	6.40	27.00	20.60	76.30
	Batouri	106	12.80	2	2	0	2.25	15.05	60.75	45.70	75.23
Total	4	372	44.80	7	7	0	6.75	51.55	211.50	159.95	75.63
Far-North	Mokolo	17	3.20	1	1	0	2.25	5.45	11.25	5.80	51.56
	Kaele	36	4.80	1	1	0	2.25	7.05	20.25	13.20	65.19
	yagoua	70	8	1	1	0	2.25	10.25	40.50	30.25	74.69
	Kousseri	103	12.80	4	4	0	2.25	15.05	58.50	43.45	74.27
Total	4	226	28.80	7	7	0	9	37.80	130.50	86.90	66.59
Littoral	Nkongsamba	239	28.80	3	3	0	2.25	31.05	135.00	103.95	77.00
	Mbanga	132	16	5	5	0	4.5	20.50	74.25	53.75	72.39
	Edéa	255	30.40	4	4	0	2.25	32.65	144.00	111.35	77.33
Total	3	626	75.20	12	12	0	9	84.20	353.25	269.05	76.16
North	Poli	98	11.20	0	0	0	0	11.20	56.25	45.05	80.09
	Guider	143	17.60	6	6	0	4.5	22.10	81.00	58.90	72.72
Total	2	241	28.80	6	6	0	4.5	33.30	137.25	103.95	75.74
Sud	Sangmelima	44	6.40	5	5	0	4.5	10.90	24.75	13.85	55.96
	Ebolowa	55	6.40	3	3	0	2.25	8.65	31.50	22.85	72.54
	Kribi	130	16	3	3	0	2.25	18.25	74.25	56.00	75.42
	Djoum	41	4.80	0	0	0	0	4.80	24.75	19.95	80.61
Total	4	270	33.60	11	11	0	9	42.60	155.25	112.65	72.56
Ouest	Mantoum	64	8.00	0	0	0	0	8.00	18	10.00	55.56
	Bagangté	40	5	0	0	0	0	4.80	10.8	6.00	55.56
	Bafang	29	4.80	3	3	0	2.25	7.05	15.86	8.81	55.56
	Foumban	46	6.40	1	1	0	2.25	8.65	19.46	10.81	55.56
Total	4	179	24.00	4	4	0	4.5	28.50	64.13	35.63	55.56
	34	3672	444.80	123	123	3	90	543.80	2053.13	1503.53	73.23
Villages at the East Region	Ndokayo Health Area	176	20.80	3	3	0	2.25	23.05	99.00	75.95	76.72
	Tongo Gandima Health Area	167	19.20	6	6	0	4.50	23.70	94.50	70.80	74.92
	Ouami Health Area	60	8	3	3	0	2.25	10.25	33.75	23.50	69.63
Total	3	403	48	12	12	0	9.00	57.00	227.25	170.25	74.92

*TB-LAMP* loop-mediated isothermal amplification assay for tuberculosis

The 3672 presumptive pulmonary TB prison cases were enrolled from 34 prisons in 8 regions of Cameroon and had an average age of 35.4 years (range: 22–62). As presented in Table 3, night sweats was the main clinical symptom observed (52%) followed by fever in 47.7% of prison cases. For the three villages (Ndokayo, Tongo-Gandima, Ouamy) in the Eastern region of Cameroon, 403 presumptive pulmonary TB cases were enrolled in the TB mass-screening campaign, the average age was 46.1 years (range: 16–90). The main clinical symptoms observed were chest pain (83.8%), weight loss (70.1%), fever (69.3%) and dyspnea (57.9%).

**Table 3:** Clinical and biomedical characteristics of presumptive cases

Characteristics	Prisons (N=3672)	Village (N= 403)
Gender		
Female	94 (2.6%)	226 (56.1%)
Age in years (mean(min.–max.))	35.4 (22–62)	46.1 (16–90)
Clinical symptoms		
Cough	3672 (100%)	29 (7.7%)
Fever	1752 (47.7%)	278 (69.3%)
Hemoptytis	813 (22.1%)	46 (11.6%)
Night sweats	1908 (52%)	253 (63.1%)
Chest pain	–	337 (83.8%)
Dyspnea	547 (14.9%)	232 (57.9%)
Weight loss	–	279 (70.1%)
Alcohol	1369 (37.3%)	64 (16%)
Smokers	1239 (33%)	10 (2.6%)

### Laboratory performance and turnaround time

Of the 4075 presumptive TB cases, 135 were laboratory confirmed (3.31%) of which 123 (3.34%) were prison cases and 12 (2.9%) were village cases (Tables 4, 5). Of the 123 TB prison cases, 03 were RR (2.36%). The three cases were from two prisons (Nkondengui and Nanga Eboko) in the Centre region (Table 4). The TAT of the new diagnostic algorithm using TB-LAMP as initial diagnosis followed by Xpert MTB/RIF compared to the diagnostic algorithm using Xpert MTB/RIF assay only is presented in Table 2. With 3672 samples, a total of 543.8 h was used for TB diagnosis with TB-LAMP and Xpert MTB/RIF assays in combination during TB mass-screening in prison compared to 2053.13 h for Xpert MTB/RIF alone. Concerning TB mass-screening in villages, 403 samples were analyzed within 57 h with the help of TB-LAMP and Xpert MTB/RIF assays performed in combination compared to 227.25 for Xpert MTB/RIF only. Our results showed that TAT was reduced to about 73.23% and 74.92%, respectively for prisons and villages with TB-LAMP as initial diagnostic tool followed by Xpert MTB/RIF.

**Table 4:** Repartition of presumptive and TB cases in prisons/region

Regions	Number of Prisons	Prison capacity or number of inmates	PTB-Presumptive cases TB-LAMP	Percentage (%)	TB-LAMP Positive	Percentage (%)	TB-LAMP Positive cases Xpert MTB/RIF	RR Xpert MTB/RIF	Percentage (%)
Adamaoua	2	1729	256	14.81	19	7.42	19	0	0.00
Centre	11	7590	1502	19.79	57	3.79	57	3	5.26
East	4	1333	372	27.91	7	1.88	7	0	0.00
Far-North	4	1619	226	13.96	7	3.10	7	0	0.00
Littoral	3	1090	626	57.43	12	1.92	12	0	0.00
North	2	589	241	40.92	6	2.49	6	0	0.00
South	4	1077	270	25.07	11	4.07	11	0	0.00
West	4	672	179	26.64	4	2.23	4	0	0.00
Total	34	15699	3672	23.39	123	3.35	123	3	2.44

*TB-LAMP* loop-mediated isothermal amplification assay for tuberculosis

**Table 5:** Repartition of presumptive and TB cases/village

Regions	Villages	Population	Screened population	TB-Presumptive case analysed with TB-LAMP	Percentage (%)	TB-LAMP Positive	Percentage (%)	TB-LAMP Positive cases analysed with Xpert MTB/RIF	Rifampicine Resistance detected with Gene Xpert MTB/RIF	Percentage (%)
East	Ndokayo Health Area	5657	234	176	75.21	3	1.70	3	0	0.00
	Tongo Gandima Health Area	19834	201	167	83.08	6	3.59	6	0	0.00
	Ouami Health Area	5120	68	60	83.24	3	5.00	3	0	0.00
Total		30611	503	403	80.12	12	2.98	12	0	0.00

*TB-LAMP* loop-mediated isothermal amplification assay for tuberculosis

### Benefit–cost analysis

The use of TB-LAMP as initial diagnostic tool followed by Xpert MTB/RIF for diagnosing TB during mass campaign showed that this algorithm is cost benefit for years 2 and 3. As shown in Table 1, about 16,934 and 19,474 USD are saved for years 2 and 3, respectively in prison and 1877 and 2158 USD are saved for years 2 and 3, respectively in village, respectively.

## Discussion

In this study, we showed that a diagnostic algorithm using TB-LAMP and Xpert MTB/RIF assays during mass screening campaign leads to early availability of diagnostic results and is cost effective. The TAT of using both TB-LAMP and Xpert MTB/RIF assays during TB mass screening was shorter than TAT of using Xpert MTB/RIF assay only. In prison and village campaigns, we observed a 3/4 reduction in the number of hours required for testing the same sample volume. This reduction in the TAT for TB confirmation in laboratory was beneficial as it allowed positive patients to be quickly isolated and placed on treatment. Therefore, this decrease in TAT for detecting MTB and early initiation of treatment would not have been possible if Xpert MTB/RIF was used only. Fundamentally, the GeneXpert equipment was introduced into clinical practice to expedite diagnosis. However in Cameroon, most TB diagnostic facilities are only equipped with the four module GeneXpert system that can process a maximum of 16 samples/day [17]. When confronted with high sample volumes like during mass screening campaigns, their clinical utility becomes questionable. For example, in 2018, when only the Xpert MTB/RIF equipment was used to test 258 sputum samples from 5 prisons at the Far North region, it took 18 days to complete laboratory analysis of the samples with the four modules instruments GeneXpert equipment. This delayed testing could be worst in laboratories that also uses the GeneXpert equipment to diagnose other diseases such as HIV and Hepatitis C than TB [25]. In such a situation, the priority of using the GeneXpert equipment would be given to the daily samples of the laboratory. Consequently, the delay of processing for samples from TB screening mass campaign could be increased. In addition, for Xpert MTB/RIF analysis, samples should be refrigerated at 2–8 °C for up to 10 days [17]. However, because of the delay in sample processing, TB laboratories have to store samples at 2–8 °C for more than 10 days during TB mass screening campaigns. This extended storage time poses a problem of adequate conservation of samples, questions the quality of those samples at the time of analysis. For this reason, in 2019 after introducing TB-LAMP in Cameroon for TB diagnosis, the NTCP recommended that TB-LAMP be used in combination with Xpert MTB/RIF to shorten the TAT of TB diagnosis during TB mass-screening campaigns that will lead to early treatment.

Our cost analysis of using TB-LAMP and Xpert MTB/RIF assays in comparison to Xpert MTB/RIF assay shows a cost saving of about 16,934 and 19,474 USD for year 2 and 3, respectively in prison and 1877 and 2158 USD for year 2 and 3, respectively in village. We used 2019 prices available in the global drug facility catalogue to estimate the cost of the different testing algorithm. That is 9.8 USD for an Xpert MTB/RIF cartridges and 8.60 USD for a TB-LAMP test [26] regarding year 1. However, on March 24, 2020, the World TB Day, the Global Drug Facility (GDF) dropped the cost of a TB-LAMP test to 6.0 USD per patient [27, 28]. Accordingly, we considered the new price of TB-LAMP test (6 USD) for years 2 and 3.

This mass screening in prisons and villages was important as 135 PTB cases that were missed by the passive screening methods were detected. Of the 15,699 prisoners screened 123 were TB positive resulting to a notification rate of 783/100000 inmates. This notification rate is about 4 times higher than the national TB rate (186/100000) (12). The higher notification rate could be due to overcrowding and poor ventilation at prison facilities [29]. Concerning villages, of the 30,611 inhabitants screened, 12 were TB positives cases resulting to a notification rate of 40/100000 inhabitants. Although this village notification rate was about 1.6 times higher than that of the East region (25/100000) where the villages are situated [10] and was 4.65 times less than the national TB rate (186/100000) (12). In addition to TB cases detected, 03 RR-TB cases were detected in prisons. This detection rate of 2.36% amongst prisoners was higher than the estimated national yearly incidence of 1.6%RR-TB among new tuberculosis cases (11). The detection of TB and RR cases supports the utility of mass screening campaigns especially in prison facilities.

According to WHO, Cameroon is not considered as a TB MDR burdened country. As such, our proposed algorithm that first seeks to detect TB cases before testing for RR is adequate, less costly and timely for mass screenings in prisons and villages with refugee camps.

This study is a secondary analysis of the data collected by the NTCP, which represent his main limitation. Two possible consequences derive from this limit; first, the cost estimation analysis did not include a sensitivity and uncertainty analysis. Sensitivity and uncertainty analysis are important as they examine the effect of changing assumptions and cost elements over time. In our cost assessment, we assumed that there will be no change in the cost of the elements considered. The unit priced for most TB reagents or consumables has remained fixed for a while now. For example, the Gene Xpert cartridge is still sold at the same price of 9.98USD since 2012 when the test was introduced for routine diagnosis in Cameroon. Secondly, we were unable to determine the actual time it took from sample collection to results for each of the mass screening campaigns. All the mass screening campaigns did mention a time for sample collection but failed to capture when results were transmitted. Our TAT analysis was solely based on the generally accepted time it takes to process a sputum sample using the equipment.

## Conclusions

This study demonstrated the advantages of the new algorithm for TB mass screening campaigns using TB-LAMP as initial diagnostic tool followed by Xpert MTB/RIF assays for all positive TB-LAMP samples. This algorithm improves early and rapid TB detection and drug resistance with respect to the TAT during TB mass-screening campaigns in prisons and villages with refugee camps. In addition, these assays used in combination were cost saving and shortens the time leading to anti-TB treatment initiation. Our study strongly acclaims the routine use of TB-LAMP and Xpert MTB/RIF assays in combination for TB diagnosis and MDR-TB which can substantially reduce the cost of the analysis from the second year of implementation. However, an adequate sample transport system should be implemented.

## Supplementary Information

**Additional file 1.** Estimation cost of each testing algorithms for three consecutive years.

## Data Availability

The datasets used and/or analysed during the current study are available from the corresponding author on reasonable request.

## Abbreviations

TB: Tuberculosis
PTB: Pulmonary TB
TAT: Turnaround time
RR: Rifampicin resistance
NTCP: National Tuberculosis Control Program
DTCs: Diagnosis and Treatment Centers
NRL-TB: National Reference Laboratory
WRD: WHO-recommended rapid diagnostic
