# Supplementary material for: A cost–benefit algorithm for rapid diagnosis of tuberculosis and rifampicin resistance detection during mass screening campaigns
Source: BMC Infect Dis. 2022 Mar 4;22:219. doi: 10.1186/s12879-022-07157-0 (PMC8895851; doi:10.1186/s12879-022-07157-0)
Supplement: Supplementary file 1 — Additional file 1. Estimation cost of each testing algorithms for three consecutive years. [file 12879_2022_7157_MOESM1_ESM.pdf]

Supplementary file

Assumptions:

1- Epidemiological situation is the same between year 1, year 2 and year 3
2- 15 % increase of sample collected per year
3- Cost per cartridge concerning Xpert is stable between year 1 and year 3; while Cost per Test concerning TB-LAMP decrease between year 1 and year 2 and is stable between year 2 and year 3
4- Both algorithm are implemented in same settings

Table 6: Cost estimate for prisons using Algorithm 1 (Testing with Gene Xpert MTB/RIF) for three years

		**Algorithm 1: Testing with Gene Xpert MTB/RIF**		
**Items**		**Year 1**	**Year 2**	**Year 3**
**GeneXpert GX-IV (4-Module System) with Desktop and 2-years warranty extension ***		**17000**	**NA**	**NA**
**Shipment**		**1000**	**NA**	**NA**
**UPS**		**1000**	**NA**	**NA**
**Printer**		**200**	**NA**	**NA**
**Air-conditioners**		**200**	**NA**	**NA**
**1-year warranty extension – GX-IV (4-Module System) ***		**NA**	**NA**	**2898**
**Consumables**		**40311.21**	**46357.89**	**53311.58**
	Cost per cartridge *	9.98	9.98	9.98
	Number of tests performed during mass screening campaign (15% increase in collected samples number per year)	3672	4222.8	4856.22
	Losses due to error/incorrect use (high estimate 10%)	367.2	422.28	485.62
	Total tests performed	4039.2	4645.08	5341.84

**HR costs (per diem)**		**27880**	**27880**	**27880**
	Technician (160 USD/prison×34 prisons)	5440	5440	5440
	Medical Doctor (160 USD/prison×34 prisons	5440	5440	5440
	Nurses (160 USD/prison)	5440	5440	5440
	Prison staff (20 USD/prison×34 prisons)	680	680	680
	TB Regional Coordinator(160 USD/prison)	5440	5440	5440
	Driver (160 USD/prison×34 prisons)	5440	5440	5440
**Total Cost**		**87591.21**	**74237.89**	**84089.58**

**References of prices: Global Drug Facility (GDF). Diagnostics Catalog 2018. available on* http://www.stoptb.org/assets/documents/gdf/Diagnostics_Catalog_2018_WEB.pdf*. NA: not applicable*

Table 7: Cost estimate for prisons using Algorithm 2 (Initial testing with TB-LAMP followed by testing with Xpert MTB/RIF) for three years

**Algorithm 2: Initial testing with TB-LAMP followed by testing with Xpert MTB/RIF**						
	**Year 1**		**Year 2**		**Year 3**	
	**HumaLoopT with 1 year warranty upon purchase**	**GeneXpert GX-IV (4-Module System) with Desktop and 2-years warranty upon purchase**	**HumaLoopT**	**GeneXpert GX-IV (4-Module System) with Desktop and 2-years warranty upon purchase**	**HumaLoop T**	**GeneXpert GX-IV (4-Module System) with Desktop**
**Items**						
**Equipement ***	**2870**	**17000**	**NA**	**NA**	**NA**	**NA**
**Shipment**	**800**	**1000**	**NA**	**NA**	**NA**	**NA**
**UPS**	**470**	**1000**	**NA**	**NA**	**NA**	**NA**

**Printer**		**NA**	**200**	**NA**	**NA**	**NA**	**NA**
**Air-conditioners**		**NA**	**200**	**NA**	**NA**	**NA**	**NA**
**1-year warranty extension – GX-IV (4-Module System) ***		**NA**	**NA**	**NA**	**NA**	**NA**	**2898**
**Maintenance with warranty extension for TB-LAMP ***		**NA**	**NA**	**1892**	**NA**	**1892**	**NA**
**Consumables**		**34737.12**	**1350.29**	**27870.48**	**1552.98**	**32051.05**	**1785.93**
	Cost per cartridge or test*	8.6	9.98	6 **	9.98	6 **	9.98
	Number of tests performed (estimate 15% increase in collected samples per year)	3672	NA	4222.8	NA	4856.22	NA
	Number of tests performed with Xpert (3.315% of TB LAMP positivity rate)	NA	123	NA	141.46	NA	162.68
	Losses due to error/incorrect use (high estimate 10%)	367.2	12.3	422.28	14.14	485.62	16.26
	Total tests performed	4039.2	135.3	4645.08	155.61	5341.84	178.95

**HR costs (per diem)**		**27880**		**27880**		**27880**	
	Technician (160 USD/prison×34 prisons)	5440		5440		5440	
	Medical Doctor (160 USD/prison×34 prisons	5440		5440		5440	
	Nurses (160 USD/prison)	5440		5440		5440	
	Prison staff (20 USD/prison×34 prisons)	680		680		680	
	TB Regional Coordinator(160 USD/prison)	5440		5440		5440	
	Driver (160 USD/prison×34 prisons)	5440		5440		5440	
**Total Cost**		**66757.12**	**20750.29**	**55750.48**	**1552.98**	**59931.05**	**4683.93**
**Total Cost per Year**		**87507.41**		**57303.46**		**64614.99**	

**References of prices: Global Drug Facility (GDF). Diagnostics Catalog 2018. available on http://www.stoptb.org/assets/documents/gdf/Diagnostics_Catalog_2018_WEB.pdf;*
***References of prices available available in the GDF diagnostics catalog. HumaLoop T*
For the prices that are indicated in Euro on the website; Exchange rate: 1€ = 1.13 $,
NA: not applicable

Table 8: Cost estimate for villages using Algorithm 1 (Testing with Gene Xpert MTB/RIF) for three years

		Algorithm 1: Testing with Gene Xpert MTB/RIF		
		**Year 1**	**Year 2**	**Year 3**
**Items**				
**GeneXpert GX-IV (4-Module System) with Desktop and 2-years warranty extension***		**17000**	**NA**	**NA**
**Shipment**		**1000**	**NA**	**NA**
**UPS**		**1000**	**NA**	**NA**
**Printer**		**200**	**NA**	**NA**
**Air-conditioners**		**200**	**NA**	**NA**
**1-year warranty extension – GX-IV (4-Module System)***		**NA**	**NA**	**2898**
**Consumables**		**4424.13**	**5087.75**	**5850.917**
	Cost per cartridge*	9.98	9.98	9.98
	Number of tests performed during mass screening campaign (15% increase in collected samples number per year)	403	463.45	532.96
	Losses due to error/incorrect use (high estimate 10%)	40.3	46.34	53.29
	Total tests performed	443.3	509.79	586.26
**HR costs (per diem)**		**2460**	**2460**	**2460**
	Technician (160 USD/village×3 villages)	480	480	480
	Medical Doctor (160 USD/village×3 villages	480	480	480
	Nurses (160 USD/village×3 villages)	480	480	480
	Community health workers (20 USD/village×3 village)	60	60	60
	TB Regional Coordinator(160 USD/village×3 village)	480	480	480

	Driver (160 USD/village×3 villages)	480	480	480
**Total Cost per year**		**26284.13**	**7547.75**	**11208.91**

**References of prices: Global Drug Facility (GDF). Diagnostics Catalog 2018. available on http://www.stoptb.org/assets/documents/gdf/Diagnostics_Catalog_2018_WEB.pdf;*
NA: not applicable

Table 9: Cost estimate for villages using Algorithm 2 (Initial testing with TB-LAMP followed by testing with Xpert MTB/RIF) for three years

**Algorithm 2: Initial testing with TB-LAMP followed by testing with Xpert MTB/RIF**						
	**Year 1**		**Year 2**		**Year 3**	
Items	**HumaLoopT with 1 year warranty upon purchase**	**GeneXpert GX-IV(4-Module System) with Desktop and 2-years warranty upon purchase**	**HumaLoopT**	**GeneXpert GX-IV (4-Module System with Desktop) and 2-years warranty upon purchase**	**HumaLoopT**	**GeneXpert GX-IV (4-Module System) with Desktop**
Equipement*	**2870**	**17000**	**NA**	**NA**	**NA**	**NA**
Shipment	**800**	**1000**	**NA**	**NA**	**NA**	**NA**
UPS	**470**	**1000**	**NA**	**NA**	**NA**	**NA**
Printer	**NA**	**200**	**NA**	**NA**	**NA**	**NA**
Air-conditioners	**NA**	**200**	**NA**	**NA**	**NA**	**NA**
1-year warranty extension – GX-IV (4-Module System)*	**NA**	**NA**	**NA**	**NA**	**NA**	**2898**
Maintenance with warranty extension for TB-LAMP*	**NA**	**NA**	**1892**	**NA**	**1892**	**NA**

Consumables		**3812.38**	**131.736**	**3058.77**	**151.61**	**3517.58**	**174.35733**
	Cost per cartridge or test*	8.6	9.98	6**	9.98	6**	9.98
	Number of performed (estimated 15% increase in collected samples per year)	403	NA	463.45	NA	532.96	NA
	Number of tests performed with Xpert (2.98% of TB LAMP positivity rate)	NA	12	NA	13.81	NA	15.88
	Losses due to error/incorrect use (high estimate 10%)	40.3	1.2	46.34	1.38	53.29	1.58
	Total tests performed	443.3	13.2	509.79	15.19	586.26	17.47
HR costs (per diem)		**2460**		**2460**		**2460**	
	Technician (160 USD/village× 3 villages)	480		480		480	
	Medical Doctor (160	480		480		480	

	USD/village×3 villages						
	Nurses (160 USD/village×3 villages)	480		480		480	
	Community health workers (20 USD/ village×3 village)	60		60		60	
	TB Regional Coordinator(160 USD/ village×3 village)	480		480		480	
	Driver (160 USD/village×3 villages)	480		480		480	
Total Cost		**10412.38**	**19531.73**	**5518.77**	**151.61**	**5977.58**	**3072.35**
Total Cost per Year		**29944.11**		**5670.38**		**9049.94**	

**References of prices: Global Drug Facility (GDF). Diagnostics Catalog 2018. available on http://www.stoptb.org/assets/documents/gdf/Diagnostics_Catalog_2018_WEB.pdf;*
***References of prices available in the GDF diagnostics catalog. HumaLoop T*
For the prices are indicated in Euro on the website. Exchange rate: 1€ = 1.13 $,
NA: not applicable
